# 5-HT_1A_ Serotonergic, α-Adrenergic and Opioidergic Receptors Mediate the Analgesic Efficacy of Vortioxetine in Mice

**DOI:** 10.3390/molecules26113242

**Published:** 2021-05-28

**Authors:** Nazlı Turan Yücel, Ümmühan Kandemir, Ümide Demir Özkay, Özgür Devrim Can

**Affiliations:** 1 Department of Pharmacology, Faculty of Pharmacy, Anadolu University, Eskişehir 26470, Turkey; nazlituran@anadolu.edu.tr (N.T.Y.); udemir@anadolu.edu.tr (Ü.D.Ö.); 2 Department of Pharmacology, Institute of Health Sciences, Anadolu University, Eskişehir 26470, Turkey; ummuhan_kandemir@hotmail.com

**Keywords:** vortioxetine, hot-plate test, tail-clip test, tail-immersion test, monoamines, opioid

## Abstract

Vortioxetine is a multimodal antidepressant drug that affects several brain neurochemicals and has the potential to induce various pharmacological effects on the central nervous system. Therefore, we investigated the centrally mediated analgesic efficacy of this drug and the mechanisms underlying this effect. Analgesic activity of vortioxetine (5, 10 and 20 mg/kg, *p.o.*) was examined by tail-clip, tail-immersion and hot-plate tests. Motor performance of animals was evaluated using Rota-rod device. Time course measurements (30–180 min) showed that vortioxetine (10 and 20 mg/kg) administrations significantly increased the response latency, percent maximum possible effect and area under the curve values in all of the nociceptive tests. These data pointed out the analgesic effect of vortioxetine on central pathways carrying acute thermal and mechanical nociceptive stimuli. Vortioxetine did not alter the motor coordination of mice indicating that the analgesic activity of this drug was specific. In mechanistic studies, pre-treatments with *p*-chlorophenylalanine (serotonin-synthesis inhibitor), NAN-190 (serotonin 5-HT_1A_ receptor antagonist), α-methyl-para-tyrosine (catecholamine-synthesis inhibitor), phentolamine (non-selective α-adrenoceptor blocker), and naloxone (non-selective opioid receptor blocker) antagonised the vortioxetine-induced analgesia. Obtained findings indicated that vortioxetine-induced analgesia is mediated by 5-HT_1A_ serotonergic, α-adrenergic and opioidergic receptors, and contributions of central serotonergic and catecholaminergic neurotransmissions are critical for this effect.

## 1. Introduction

Vortioxetine, an antidepressant drug, was licensed by the Food and Drug Administration (FDA) in September 2013 and the European Medicines Agency (EMA) in October 2013. This drug has been approved for treating major depressive disorder in adults under the trade names Brintellix^®^ and Trintellix^®^ [1,2]. Vortioxetine is a serotonergic modulator with antagonistic effects on serotonergic 5-HT_3_, 5-HT_7_, and 5-HT_1D_, agonistic effects on serotonergic 5-HT_1A_, and partial agonistic effects on serotonergic 5-HT_1B_ receptor subtypes. It also has a potent inhibitory effect on serotonin transporters [2].

Vortioxetine alters neurotransmitter levels in several brain regions. In microdialysis experiments, this drug was found to enhance extracellular serotonin, dopamine, and noradrenaline levels in the medial prefrontal cortex and ventral hippocampus of the rat brain [3]. Moreover acute vortioxetine administration increases serotonin levels in the nucleus accumbens [4]. The vortioxetine-induced increase in neurotransmitter levels is associated with the 5-HT_3_ receptor antagonistic and 5-HT_1A_ agonistic activities of this drug because these receptors are involved in regulating neurotransmitter release in multiple brain regions [5]. In addition to the serotonergic and catecholaminergic systems, vortioxetine also affects some other neurotransmitter systems in the brain. This drug has been suggested to increase extracellular acetylcholine and histamine levels in the rat medial prefrontal cortex [6] and modulate GABAergic and glutamatergic neurotransmissions in the brain [7].

As a drug affecting several brain neurochemicals, vortioxetine is expected to have a broad pharmacological activity spectrum in the central nervous system (CNS). Recent studies have shown that this molecule does indeed have anxiolytic [8], anti-panic [9], cognitive enhancing [8] and anti-epileptic [10] activities in association with the CNS. Moreover, as an antidepressant drug, vortioxetine has also the potential to block nociceptive signals by enhancing the pain suppression pathways of the CNS [11,12]. This knowledge suggests that a multimodal antidepressant drug as vortioxetine could has an analgesic activity mediated by central mechanisms. However, the analgesic efficacy of this drug on acute mechanical and thermal nociceptive stimuli together with the underlying pharmacological mechanisms has not been demonstrated, so far. Therefore, in this study, we investigated whether vortioxetine has central analgesic activity. Moreover, possible involvements of serotonergic, catecholaminergic and opioidergic systems in the analgesic effect of this drug were elucidated by mechanism studies performed with *p*-chlorophenylalanine (PCPA, serotonin-synthesis inhibitor), NAN-190 (serotonin 5-HT_1A_ receptor antagonist), α-methyl-para-tyrosine (AMPT, catecholamine-synthesis inhibitor), phentolamine (non-selective α-adrenoceptor blocker), propranolol (non-selective β-adrenoceptor blocker), and naloxone (non-selective opioid receptor blocker).

## 2. Results

### 2.1. Motor Coordination

#### Rota-Rod Test

**Figure 1:**
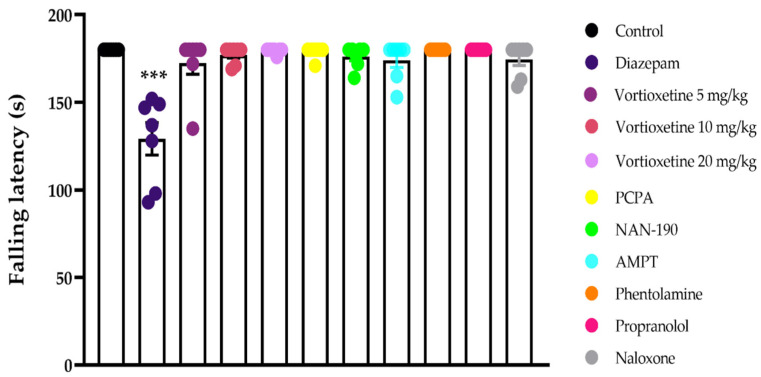
The effects of diazepam (2 mg/kg, *p.o.*), vortioxetine (5, 10 and 20 mg/kg, *p.o.*), PCPA (100 mg/kg, 4 day, *i.p.*), NAN-190 (0.5 mg/kg, *i.p.*), AMPT (100 mg/kg, *i.p.*), phentolamine (4 mg/kg, *i.p.*), propranolol (2 mg/kg, *i.p.*), and naloxone (5.48 mg/kg, *i.p.*) administrations on motor coordination parameters of mice in the Rota-Rod Test. Significant difference against control group *** *p* < 0.001, One way ANOVA, post-hoc Tukey test, n = 7.

The effects of acute administrations of vortioxetine (5, 10 and 20 mg/kg), diazepam, PCPA, NAN-190, AMPT, phentolamine, propranolol, and naloxone on the falling latencies of the mice, as assessed using the Rota-rod device, are shown in Figure 1 [F (10, 66) = 14.48, *p* < 0.001]. Tukey’s HSD test for multiple comparisons showed that the motor-coordination parameters of the mice administered vortioxetine, PCPA, NAN-190, AMPT, phentolamine, propranolol, and naloxone did not significantly differ from those of the control group mice. Diazepam at 2 mg/kg dose impaired the motor performances of mice, as expected.

### 2.2. Analgesic Activity

#### 2.2.1. Analgesic Effect of Vortioxetine on Mechanical Noxious Stimuli-Induced Pain Behavior

**Figure 2:**
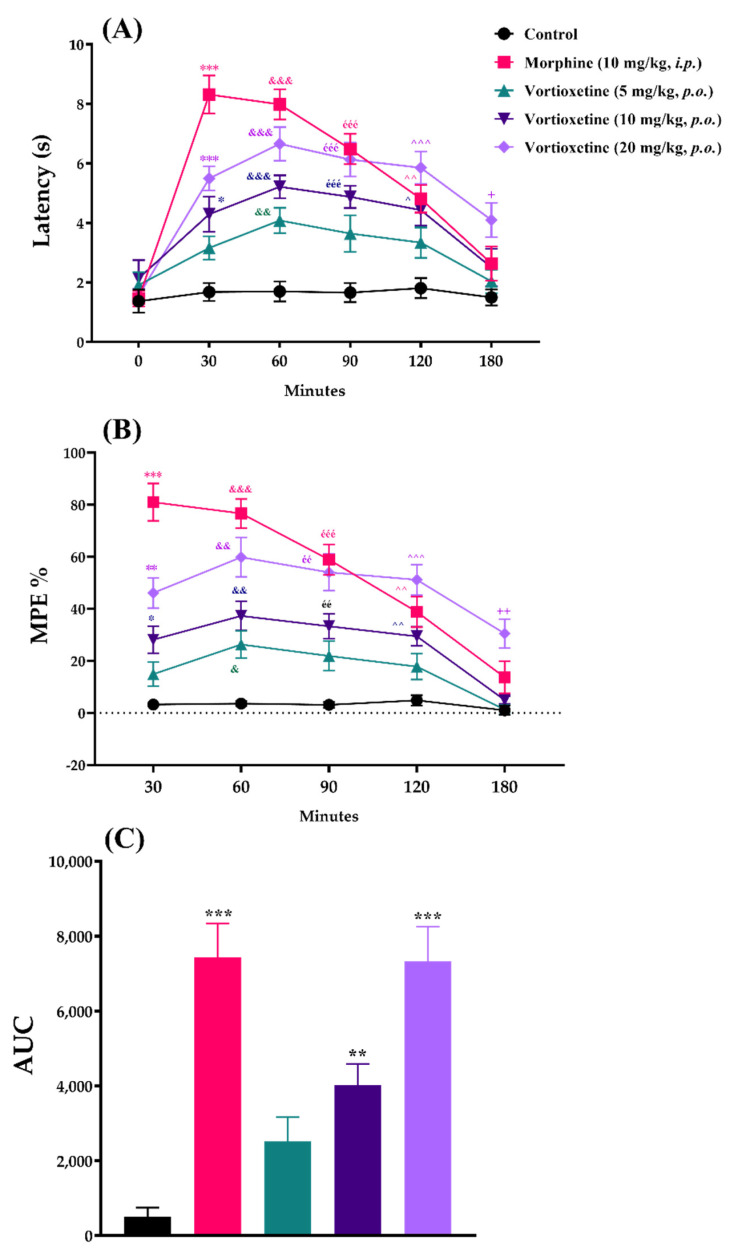
The effects of morphine (10 mg/kg, *i.p.*) and vortioxetine (5, 10 and 20 mg/kg, *p.o.*) administrations on latency (**A**), MPE% (**B**) and AUC (**C**) values of mice in the tail-clip test (mechanical nociception). (**A**) and (**B**): Significant difference against control group at 30th min * *p* < 0.05, ** *p* < 0.01, *** *p* < 0.001; at 60th min ^&^ *p* < 0.05, ^&&^ *p* < 0.01, ^&&&^ *p* < 0.001; at 90th min ^éé^ *p* < 0.01, ^ééé^ *p* < 0.001; at 120th min ^^^ *p* < 0.05, ^^^^ *p* < 0.01, ^^^^^ *p* < 0.001; at 180th min ^+^ *p* < 0.05; ^++^ *p* < 0.01. Two-way repeated ANOVA, post-hoc Tukey test, n = 7. (**C**): Significant difference against control group ** *p* < 0.01, *** *p* < 0.001; One-way ANOVA, post-hoc Tukey test, n = 7.

The effects of acute vortioxetine (5, 10 and 20 mg/kg) administrations on response latency, MPE% and AUC values obtained from the tail-clip tests are shown in Figure 2.

Two-way repeated ANOVA analysis indicated that both of the treatment ([F (4, 30) = 16.95, *p* < 0.001]) and time ([F (3.084, 92.51) = 83.55, *p* < 0.001]) factors affected the response latency values in the tail-clip tests. There was a significant interaction between treatment and time factors ([F (20, 150) = 11.33, *p* < 0.001]) (Figure 2A). Treatment ([F (4, 30) = 42.94, *p* < 0.001]) and time ([F (2.879, 86.37) = 39.47, *p* < 0.001]) factors also affected the MPE% values in the same test with a significant interaction between them ([F (16, 120) = 6.85, *p* < 0.001]) (Figure 2B). Besides, vortioxetine administrated at 10 mg/kg (*p* < 0.01) and 20 mg/kg (*p* < 0.001) doses caused significant increase in the AUC values compared to the control group ([F (4, 150) = 18.54, *p* < 0.001]) (Figure 2C).

#### 2.2.2. Spinally Mediated Analgesic Effect of Vortioxetine on Thermal Noxious Stimuli-Induced Pain Behavior

**Figure 3:**
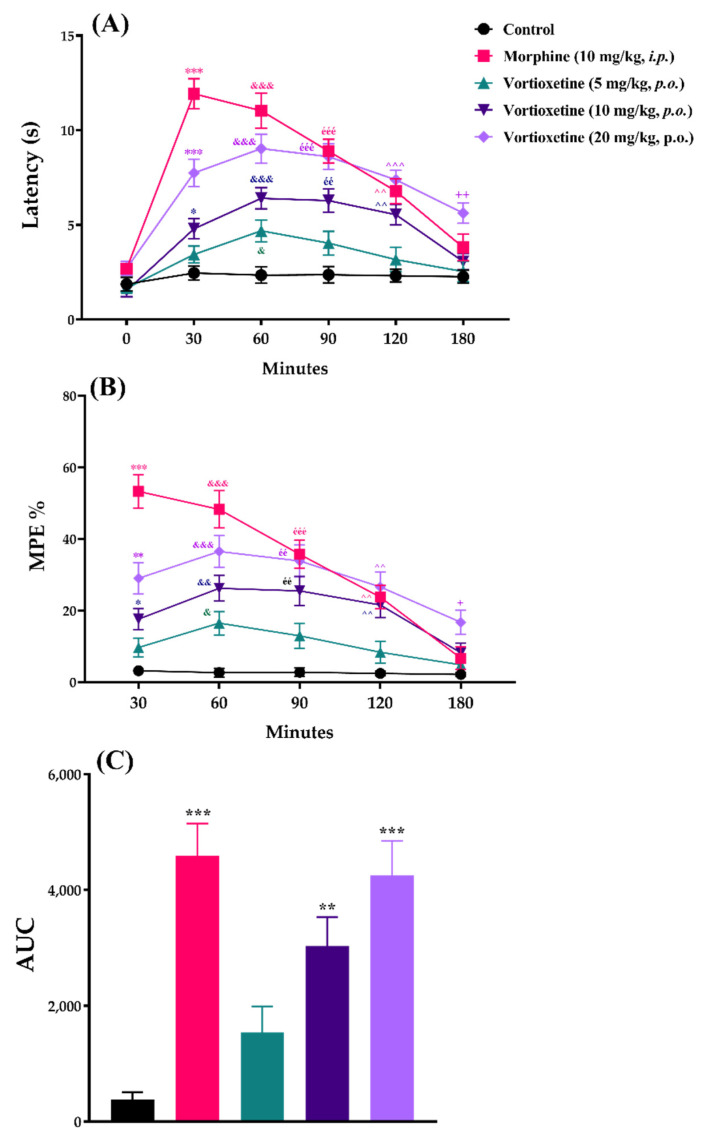
The effects of morphine (10 mg/kg, *i.p.*) and vortioxetine (5, 10 and 20 mg/kg, *p.o.*) administrations on latency (**A**), MPE% (**B**) and AUC (**C**) values of mice in the tail-immersion test (thermal nociception). (**A**) and (**B**): Significant difference against control group at 30th min * *p* < 0.05, ** *p* < 0.01, *** *p* < 0.001; at 60th min ^&^ *p* < 0.05, ^&&^ *p* < 0.01, ^&&&^ *p* < 0.001; at 90th min ^éé^ *p* < 0.01; ^ééé^ *p* < 0.001 at 120th min ^^^^ *p* < 0.01, ^^^^^ *p* < 0.001; at 180th min ^+^ *p* < 0.05, ^++^ *p* < 0.01. Two-way repeated ANOVA, post-hoc Tukey test, n = 7. (**C**): Significant difference against control group ** *p* < 0.01, *** *p* < 0.001; One-way ANOVA, post-hoc Tukey test, n = 7.

The effects of acute vortioxetine (5, 10 and 20 mg/kg) administration on response latency, MPE% and AUC values obtained from the tail-immersion tests are shown in Figure 3.

Two-way repeated ANOVA analysis indicated that both of the treatment ([F (4, 30) = 51.42, *p* < 0.001]) and time ([F (3.154, 94.63) = 64.26, *p* < 0.001]) factors affected the response latency values in the tail-immersion tests. There was a significant interaction between treatment and time factors ([F (20, 150) = 8.925, *p* < 0.001]) (Figure 3A). Treatment ([F (4, 30) = 38.0, *p* < 0.001]) and time ([F (2.858, 85.74) = 29.5, *p* < 0.001]) factors also affected the MPE% values in the same test with a significant interaction between them ([F (16, 120) = 6.716, *p* < 0.001]) (Figure 3B). Besides, vortioxetine administrated at 10 mg/kg (*p* < 0.01) and 20 mg/kg (*p* < 0.001) doses caused significant increase in the AUC values compared to the control group ([F (4, 150) = 13.98, *p* < 0.001]) (Figure 3C).

#### 2.2.3. Supraspinally Mediated Analgesic Effect of Vortioxetine on Thermal Noxious Stimuli-Induced Pain Behavior

**Figure 4:**
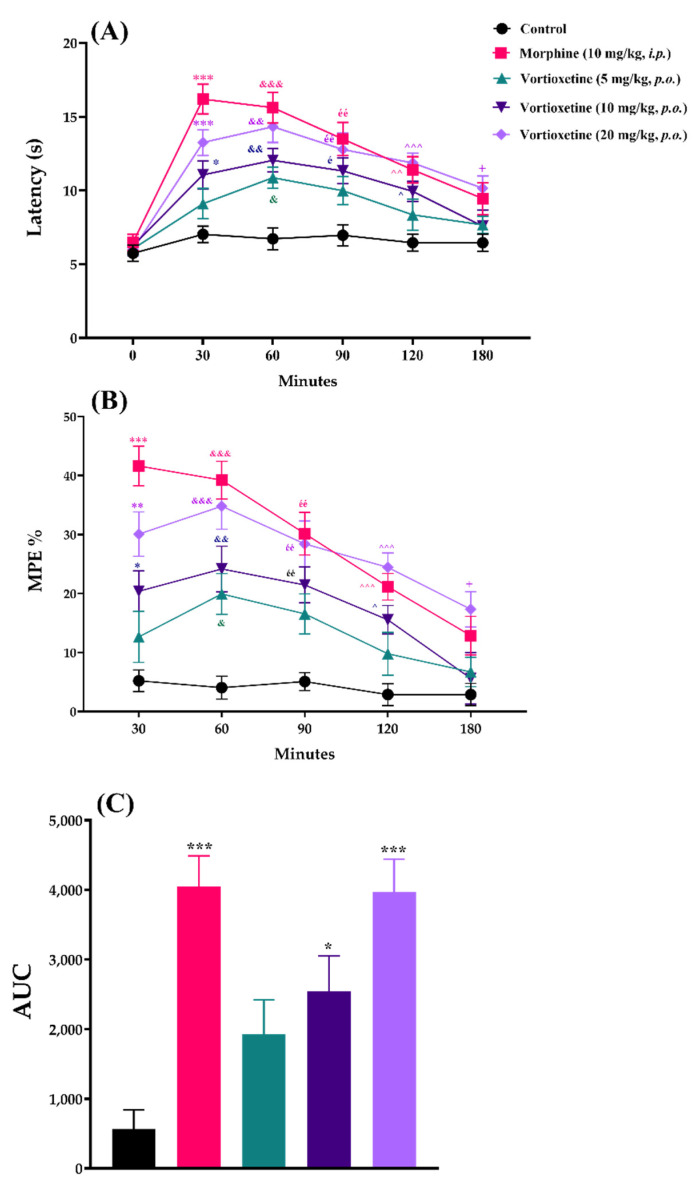
The effects of morphine (10 mg/kg, *i.p.*) and vortioxetine (5, 10 and 20 mg/kg, *p.o.*) administrations on latency (**A**), MPE% (**B**) and AUC (**C**) values of mice in the hot-plate test (thermal nociception). (**A**) and (**B**): Significant difference against control group at 30th min * *p* < 0.05, ** *p* < 0.01, *** *p* < 0.001; at 60th min ^&^ *p* < 0.05, ^&&^ *p* < 0.01, ^&&&^ *p* < 0.001; at 90th min ^é^ *p* < 0.05, ^éé^ *p* < 0.01; at 120th min ^^^ *p* < 0.05, ^^^^ *p* < 0.01, ^^^^^ *p* < 0.001; at 180th min ^+^ *p* < 0.05. Two-way repeated ANOVA, post-hoc Tukey test, n = 7. (**C**): Significant difference against control group * *p* < 0.05, *** *p* < 0.001; One-way ANOVA, post-hoc Tukey test, n = 7.

The effects of acute vortioxetine (5, 10 and 20 mg/kg) administration on response latency, MPE% and AUC values obtained from the hot-plate tests are shown in Figure 4.

Two-way repeated ANOVA analysis indicated that both of the treatment ([F (4, 30) = 12.53, *p* < 0.001]) and time ([F (3.958, 118.8) = 68.46, *p* < 0.001]) factors affected the response latency values in the hot-plate tests. There was a significant interaction between treatment and time factors ([F (20, 150) = 5.304, *p* < 0.001]) (Figure 4A). Treatment ([F (4, 30) = 23.57, *p* < 0.001]) and time ([F (3.366, 101.0) = 28.08, *p* < 0.001]) factors also affected the MPE% values in the same test with a significant interaction between them ([F (16, 120) = 2.986, *p* < 0.001]) (Figure 4B). Besides, vortioxetine administrated at 10 mg/kg (*p* < 0.05) and 20 mg/kg (*p* < 0.001) doses caused significant increase in the AUC values compared to the control group ([F (4, 150) = 10.71, *p* < 0.001]) (Figure 4C).

In all of the three nociceptive tests, results of the multiple comparison analysis showed that vortioxetine at its 20 mg/kg dose, significantly increased the response latencies and MPE% values with respect to the corresponding control levels, in all of the time points. Although this drug showed significant efficacy up to 180 min when administered at a dose of 10 mg/kg, it was effective only in the 60th minute at a dose of 5 mg/kg. Reference drug morphine (10 mg/kg, *i.p.*) was exhibited its analgesic effect, as expected (Figure 2, Figure 3 and Figure 4).

### 2.3. Mechanistic Studies

After the acute analgesic efficacy profile of vortioxetine was determined, mechanistic studies were conducted to elucidate the mechanism of this effect. In time course studies conducted with this drug, it was observed that vortioxetine showed its highest activity in the 60th minute at every dose administrated; then mechanistic studies were carried out by comparing MPE% values at 60th minute. This drug was administrated at 10 mg/kg dose in the mechanistic studies, since there were no statistically significant difference between the MPE% values of 10 and 20 mg/kg doses 60 min after the administrations.

#### 2.3.1. Involvement of Serotonergic System in the Analgesic Effect of Vortioxetine

**Figure 5:**
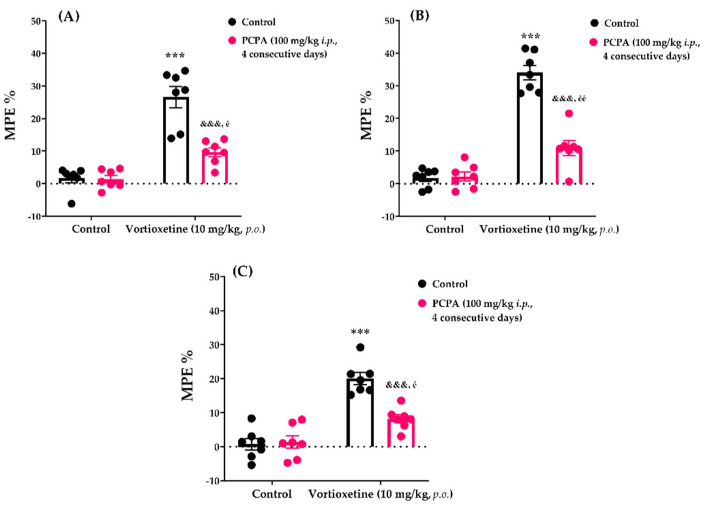
Involvement of serotonergic system in the analgesic effects of vortioxetine in the tail-clip (**A**), tail-immersion (**B**) and hot-plate (**C**) tests. Significant difference against vehicle treated control groups *** *p* < 0.001; Significant difference against vortioxetine administrated control groups ^&&&^ *p* < 0.001; Significant difference against PCPA administrated control groups ^é^ *p* < 0.05, ^éé^ *p* < 0.01. Two-way ANOVA, post-hoc Bonferroni test, n = 7.

The effects of PCPA pre-treatment on vortioxetine (10 mg/kg)-induced analgesic responses in the tail-clip, tail-immersion and hot-plate tests are shown in Figure 5A, Figure 5B and Figure 5C, respectively.

Two-way ANOVA indicated that the treatment (tail-clip test: [F (1, 24) = 126.0, *p* < 0.001]; tail-immersion test: [F (1, 24) = 71.62, *p* < 0.001]; hot-plate test: [F (1, 24) = 63.30, *p* < 0.001]) and antagonist (tail-clip test: [F (1, 24) = 38.51, *p* < 0.001]; tail-immersion test: [F (1, 24) = 19.26, *p* < 0.001]; hot-plate test: [F (1, 24) = 11.77, *p* < 0.01]) factors affected the MPE% values. Furthermore, there was a statistically significant interaction (tail-clip test: [F (1, 24) = 41.50, *p* < 0.001]; tail-immersion test: [F (1, 24) = 18.09, *p* < 0.001]; hot-plate test: [F (1, 24) = 14.40, *p* < 0.001]) between these two factors. Bonferroni analysis revealed that administration of PCPA at 100 mg/kg for 4 consecutive days antagonized the analgesic activity of vortioxetine in the tail-clip (*p* < 0.001), tail-immersion (*p* < 0.001) and hot-plate (*p* < 0.001) tests.

#### 2.3.2. Involvement of Serotonergic 5HT_1A_ Receptors in the Analgesic Effect of Vortioxetine

**Figure 6:**
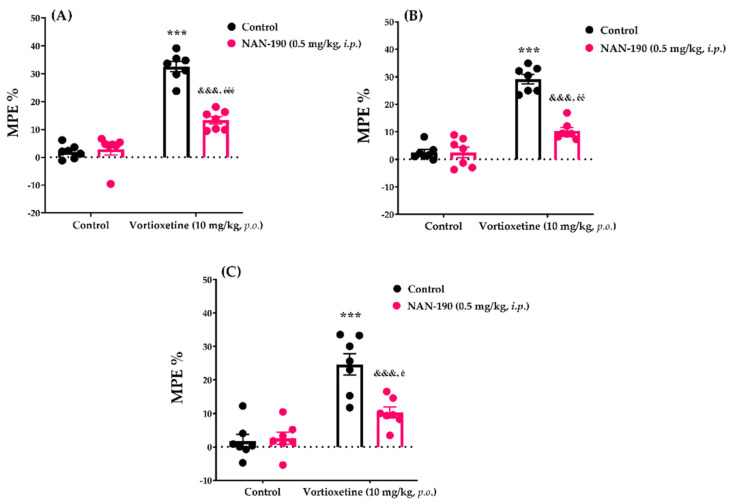
Involvement of serotonergic 5HT_1A_ receptors in the antinociceptive effects of vortiox-etine in the tail-clip (**A**), tail-immersion (**B**) and hot-plate (**C**) tests. Significant difference against vehicle treated control groups *** *p* < 0.001; Significant difference against vortioxetine administrated control groups ^&&&^ *p* < 0.001; Significant difference against NAN-190 administrated control groups ^é^ *p* < 0.05, ^éé^ *p* < 0.01, ^ééé^ *p* < 0.001. Two-way ANOVA, post-hoc Bonferroni test, n = 7.

The effects of NAN-190 pre-treatment on vortioxetine (10 mg/kg)-induced analgesic responses in the tail-clip, tail-immersion and hot-plate tests are shown in Figure 6A, Figure 6B and Figure 6C respectively. Two-way ANOVA indicated that the treatment (tail-clip test: [F (1, 24) = 160.9, *p* < 0.001]; tail-immersion test: [F (1, 24) = 126.3, *p* < 0.001]; hot-plate test: [F (1, 24) = 46.00, *p* < 0.001]) and antagonist (tail-clip test: [F (1, 24) = 32.21, *p* < 0.001]; tail-immersion test: [F (1, 24) = 37.33, *p* < 0.001]; hot-plate test: F (1, 24) = 9.03, *p* < 0.01]) factors affected the MPE% values of mice. Furthermore, there was a statistically significant interaction (tail-clip test: [F (1, 24) = 38.86, *p* < 0.001]; tail-immersion test: [F (1, 24) = 37.46, *p* < 0.001]; hot-plate test: [F (1, 24) = 11.39, *p* < 0.01]) between these two factors. Bonferroni analysis revealed that NAN-190 antagonized the analgesic activity of vortioxetine in tail-clip (*p* < 0.001), tail-immersion (*p* < 0.001) and hot-plate (*p* < 0.001) tests.

#### 2.3.3. Involvement of Catecholaminergic System in the Analgesic Effect of Vortioxetine

**Figure 7:**
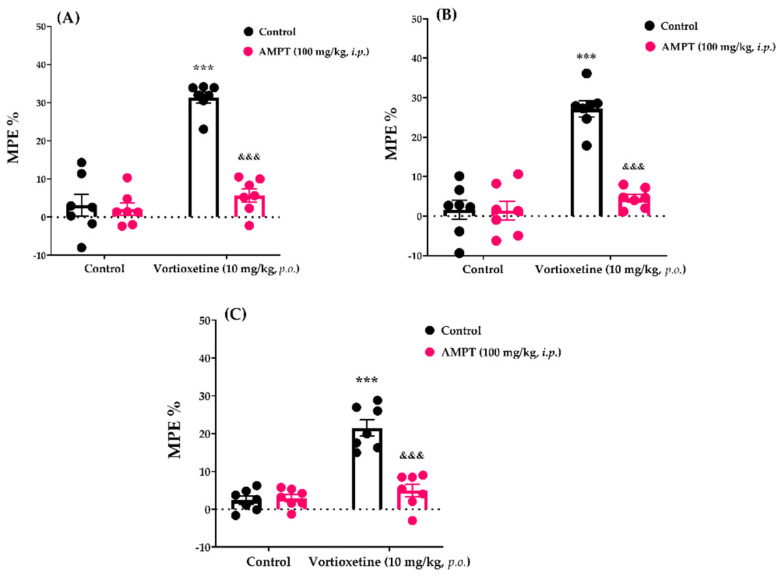
Involvement of catecholaminergic system in the analgesic effects of vortioxetine in the tail-clip (**A**), tail-immersion (**B**) and hot-plate (**C**) tests. Significant difference against vehicle treated control groups *** *p* < 0.001; Significant difference against vortioxetine administrated control groups ^&&&^ *p* < 0.001. Two-way ANOVA, post-hoc Bonferroni test, n = 7.

The effects of AMPT pre-treatment on the vortioxetine (10 mg/kg)-induced analgesic responses in the tail-clip, tail-immersion and hot-plate tests are shown in Figure 7A, Figure 7B and Figure 7C, respectively. Two-way ANOVA indicated that the treatment (tail-clip test: [F (1, 24) = 62.75, *p* < 0.001]; tail-immersion test: [F (1, 24) = 49.62, *p* < 0.001]; hot-plate test: [F (1, 24) = 48.15, *p* < 0.001]) and antagonist (tail-clip test: [F (1, 24) = 44.15, *p* < 0.001]; tail-immersion test: [F (1, 24) = 31.41, *p* < 0.001]; hot-plate test: F (1, 24) = 27.83, *p* < 0.001]) factors affected the MPE% values. Furthermore, there was a statistically significant interaction (tail-clip test: [F (1, 24) = 37.78, *p* < 0.001]; tail-immersion test: [F (1, 24) = 30.26, *p* < 0.001]; hot-plate test: [F (1, 24) = 31.61, *p* < 0.001]) between these two factors. Bonferroni analysis revealed that AMPT antagonized the analgesic activity of vortioxetine in the tail-clip (*p* < 0.001), tail-immersion (*p* < 0.001) and hot-plate (*p* < 0.001) tests.

#### 2.3.4. Involvement of α-Adrenergic Receptors in the Analgesic Effect of Vortioxetine

**Figure 8:**
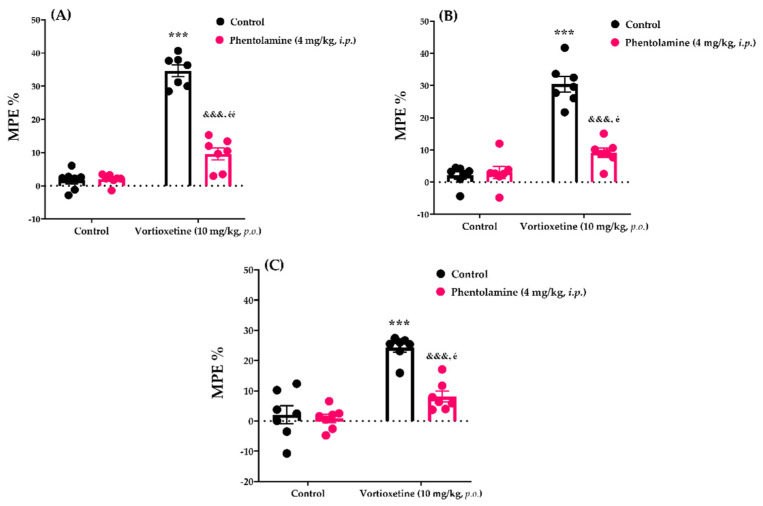
Involvement of α-adrenergic receptors in the analgesic effects of vortioxetine in the tail-clip (**A**), tail-immersion (**B**) and hot-plate (**C**) tests. Significant difference against vehicle treated control groups *** *p* < 0.001; Significant difference against vortioxetine administrated control groups ^&&&^ *p* < 0.001; Significant difference against phentolamine administrated control groups ^é^ *p* < 0.05, ^éé^ *p* < 0.01. Two-way ANOVA, post-hoc Bonferroni test, n = 7.

The effects of phentolamine pre-treatment on vortioxetine (10 mg/kg)-induced analgesic responses in the tail-clip, tail-immersion and hot-plate tests are shown in Figure 8A, Figure 8B and Figure 8C, respectively. Two-way ANOVA indicated that the treatment (tail-clip test: [F (1, 24) = 208.6, *p* < 0.001]; tail-immersion test: [F (1, 24) = 94.27, *p* < 0.001]; hot-plate test: [F (1, 24) = 53.11, *p* < 0.001]) and antagonist (tail-clip test: [F (1, 24) = 77.62, *p* < 0.001]; tail-immersion test: [F (1, 24) = 32.77, *p* < 0.001]; hot-plate test: F (1, 24) = 18.71, *p* < 0.001]) factors affected the MPE% values. Furthermore, there was a statistically significant interaction (tail-clip test: [F (1, 24) = 80.75, *p* < 0.001]; tail-immersion test: [F (1, 24) = 39.06, *p* < 0.001]; hot-plate test: [F (1, 24) = 13.75, *p* < 0.01]) between these two factors. Bonferroni analysis revealed that administration of phentolamine at 4 mg/kg (*i.p.*) led to antagonism of the analgesic activity of vortioxetine in the tail-clip (*p* < 0.001), tail-immersion (*p* < 0.001) and hot-plate (*p* < 0.001) tests.

#### 2.3.5. Involvement of β-Adrenergic Receptors in the Analgesic Effect of Vortioxetine

**Figure 9:**
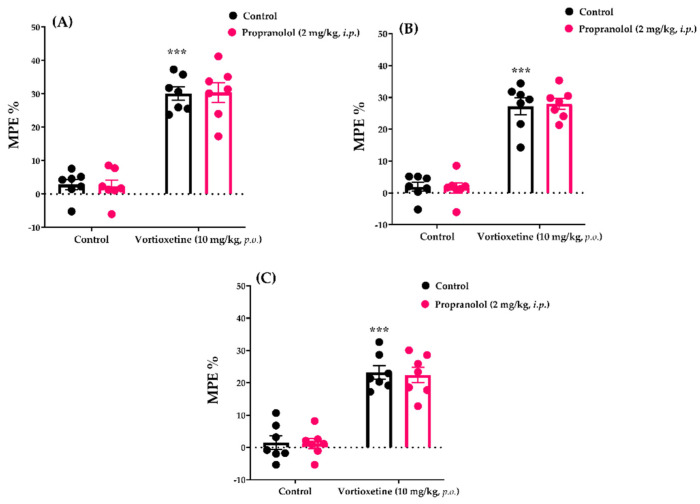
Involvement of β-adrenergic receptors in the analgesic effects of vortioxetine in the tail-clip (**A**), tail-immersion (**B**) and hot-plate (**C**) tests. Significant difference against vehicle treated control groups *** *p* < 0.001. Two-way ANOVA, post-hoc Bonferroni test, n = 7.

The effects of propranolol pre-treatment on the vortioxetine (10 mg/kg)-induced analgesic responses in tail-clip, tail-immersion and hot-plate tests are shown in Figure 9A, Figure 9B and Figure 9C, respectively.

Two-way ANOVA indicated that the treatment (tail-clip test: [F (1, 24) = 166.4, *p* < 0.001]; tail-immersion: [F (1, 24) = 185.3, *p* < 0.001]; hot-plate: [F (1, 24) = 108.5, *p* < 0.001]) factors affected the MPE% values, whereas the antagonist factor did not (tail-clip test: [F (1, 24) = 0.002, *p* > 0.05]; tail-immersion: [F (1, 24) = 0.01, *p* > 0.05]; hot-plate: F (1, 24) = 0.07, *p* > 0.05]). In addition, there was no significant interaction (tail-clip test: [F (1, 24) = 0.03, *p* > 0.05]; tail-immersion: [F (1, 24) = 0.09, *p* > 0.05]; hot-plate: [F (1, 24) = 0.01, *p* > 0.05]) between these two factors.

Bonferroni analysis revealed that administration of propranolol at 2 mg/kg (*i.p.*) did not cause antagonism of the analgesic activity of vortioxetine in the tail-clip (*p* > 0.05), tail-immersion (*p* > 0.05) and hot-plate tests (*p* > 0.05).

#### 2.3.6. Involvement of Opioid Receptors in the Analgesic Effect of Vortioxetine

**Figure 10:**
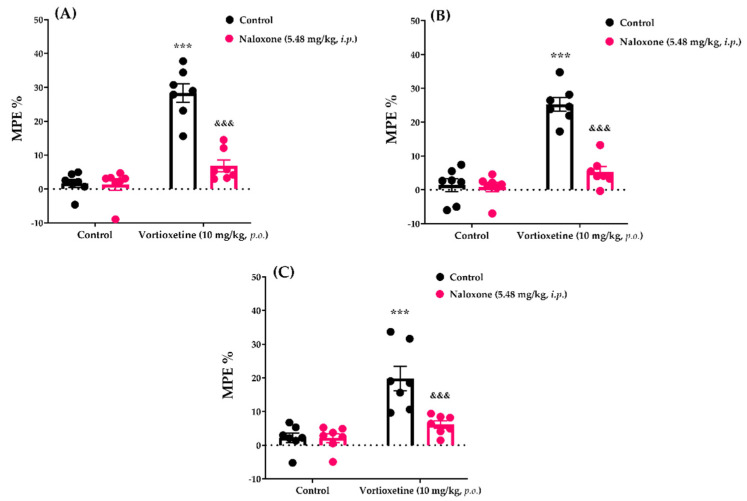
The effects of naloxone pretreatments on vortioxetine-induced analgesia in the tail-clip (**A**), tail-immersion (**B**) and hot-plate (**C**) tests. Significant difference against vehicle treated control groups *** *p* < 0.001; Significant difference against vortioxetine administrated control groups ^&&&^ *p* < 0.001. Two-way ANOVA, post-hoc Bonferroni test, n = 7.

The effects of naloxone pre-treatment on vortioxetine (10 mg/kg)-induced analgesic responses in the tail-clip, tail-immersion and hot-plate tests are shown Figure 10A, Figure 10B and Figure 10C, respectively. Two-way ANOVA indicated that the treatment (tail-clip test: [F (1, 24) = 68.51, *p* < 0.001]; tail-immersion test: [F (1, 24) = 65.43, *p* < 0.001]; hot-plate test: [F (1, 24) = 26.23, *p* < 0.001]) and antagonist (tail-clip test: [F (1, 24) = 31.57, *p* < 0.001]; tail-immersion test: [F (1, 24) = 34.47, *p* < 0.001]; hot-plate test: [F (1, 24) = 10.65, *p* < 0.01]) factors affected the MPE% values. Furthermore, there was a statistically significant interaction (tail-clip test: [F (1, 24) = 29.63, *p* < 0.001]; tail-immersion test: [F (1, 24) = 30.73, *p* < 0.001]; hot-plate test: [F (1, 24) = 10.36, *p* < 0.01]) between these two factors. Multiple comparison Bonferroni analysis revealed that pre-treatment with naloxone (5.48 mg/kg; single dose) led to antagonism of the analgesic activity of vortioxetine in the tail-clip (*p* < 0.001), tail-immersion (*p* < 0.001) and hot-plate (*p* < 0.001) tests.

## 3. Discussion

In the current study, we investigated the potential analgesic efficacy of vortioxetine based on its multimodal activity profile in the CNS. The use of agents that disrupt motor activity in experimental animals can lead to misleading results in pain experiments [13,14]. Therefore, before investigating the analgesic efficacy of vortioxetine, the possible effect of this drug on the motor coordination of the mice was investigated using Rota-rod tests. We found that vortioxetine did not significantly affect motor activities at any of the doses administered (5, 10 and 20 mg/kg). These findings, in line with previous reports [15,16], is important because it showed that the data obtained from further analgesic activity tests were specific.

The analgesic activity of vortioxetine was evaluated by the tail-clip, tail-immersion and hot-plate tests. In the tail-clip tests, animals administered vortioxetine at 10 and 20 mg/kg had significantly longer reaction time than saline-administered control mice. Moreover, MPE% and AUC values were also significantly higher with respect to the control groups. 5 mg/kg dose of this drug was only effective at 60th minute (Figure 2). In the tail-clip method, the clamp-biting reaction of animals is known to be associated with spinal transmission of nociception [14,17]. Therefore, our findings suggest that the analgesic activity of vortioxetine is related to its effect on spinal nociceptive pathways that carry painful mechanical stimuli.

The tail-immersion method [18] was another procedure used to evaluate spinally mediated acute analgesic effect, in this study. In these tests, vortioxetine at doses of 10 and 20 mg/kg significantly increased the reaction times of the mice as well as the MPE% and AUC values compared to the saline-treated control groups. A 5 mg/kg dose was effective only at the 60th minute (Figure 3). Findings from tail-immersion tests showed that vortioxetine affects also spinal nociceptive pathways that carry thermal painful stimuli along with mechanical ones.

Similar to the findings for the tail-clip and tail-immersion tests, in the hot-plate tests, vortioxetine administrated at 10 and 20 mg/kg prolonged the response latencies of mice and enhanced the related MPE% and AUC values, significantly (Figure 4). However, different from the tail-clip and tail-immersion tests, which measure spinal reflexes, the nociceptive stimulus-induced paw-licking or jumping behaviors in hot-plate test are known to be related to the supraspinal pathways [14,17]. Therefore, it can be suggested that vortioxetine-induced analgesia is mediated by supraspinal mechanisms in addition to spinal ones.

In parallel to our results, 10 mg/kg/day *i.p.* injection of vortioxetine for 27 days has been shown to reduce tactile allodynia in mice with chronic constriction damage [16]. Similarly, in oxaliplatin-induced neuropathic mice, vortioxetine has been shown to reduce mechanical allodynia in the Von Frey test and cold allodynia in acetone tests at 1–10 mg/kg *p.o.* doses [19]. Moreover, studies investigating the efficacy of vortioxetine on chemically-induced acute pain have revealed that this drug is effective in both phases of formalin test [20,21]. Our study is different from these previous studies since we used direct mechanical and thermal nociceptive stimulus (but not chemical) to induce pain instead of allodynia. Moreover, we administrated vortioxetine at 20 mg/kg *p.o.* dose, which has not been tested in nociceptive setups before. On the other hand, the only comparable study was conducted by Mørk et al., who tested the acute effect of vortioxetine (2.5, 5, and 10 mg/kg, *s.c.*) in hot-plate method. The results of the mentioned paper, showing that the 10 mg/kg dose of vortioxetine causes a significant increase in the reaction times of the animals, support our findings in this study [6].

After presenting the acute analgesic efficacy of vortioxetine against mechanical and thermal nociceptive stimuli; the possible mechanisms underlying this analgesic activity were examined, in this study. As serotonergic, catecholaminergic and opioidergic systems play critical roles in the nociception and analgesia processes of the CNS [22,23,24], we searched the possible contribution of these systems to the analgesic effect of vortioxetine.

The participation of the endogenous serotonergic system in the analgesic effect of vortioxetine was examined using PCPA, a serotonin-depleting agent. PCPA administration (100 mg/kg; four consecutive days) was previously found to block serotonin synthesis by inhibiting the tryptophan hydroxylase enzyme; furthermore, this process depleted 60–90% of the endogenous serotonin stores in the CNS without affecting the central noradrenaline and dopamine levels [25]. Results of the nociceptive tests indicated that PCPA pre-treatment antagonized the analgesic activity of vortioxetine (Figure 5). These findings revealed that the central serotonin level, which is probably modulated by the serotonin reuptake inhibitory effect of this drug [2], is critical for vortioxetine-induced analgesia.

Serotonin has extremely complex effects on pain transmission and modulation; it can induce both analgesic and pronociceptive effects in the CNS depending on receptor availability and affinity, ligand concentration, and the neural network involved [26]. 5-HT_1A_ subtypes of serotonergic receptors have been shown to densely localized in the dorsal horn of the spinal cord and in brain regions that related to pain processing or modulation [27,28,29]. These receptor subtypes have been revealed to play critical roles in analgesia [30,31,32,33] and ligands that activate them have been demonstrated to possess notable analgesic activities [34,35,36,37,38]. Based on the prominent agonistic effect of vortioxetine on 5-HT_1A_ receptors, we examined the possible participation of this receptor subtype to the analgesic effect of this drug. The fact that pre-treatment with NAN-190, a selective 5-HT_1A_ receptor antagonist, attenuated the analgesic activity of vortioxetine (Figure 6), suggests that 5-HT_1A_ receptors contribute to the acute analgesic effect of this drug. On the other hand, along with the 5-HT_1A_, possible modulatory effects of other serotonergic receptors, especially the 5-HT_1B_, 5-HT_1D_, 5-HT_3_, and 5-HT_7_ subtypes [23,27], on vortioxetine-induced analgesia should be clarified in further studies.

We investigated the involvement of the catecholaminergic system in the analgesic activity of vortioxetine by using AMPT, a catecholamine-depleting agent. AMPT pre-treatment at a dose of 100 mg/kg inhibits tyrosine hydroxylase, a rate-limiting enzyme in noradrenaline and dopamine syntheses [39]. AMPT pre-treatment lowers central dopamine and noradrenaline levels by 57% and 53%, respectively, but does not affect central serotonin levels [40]. The AMPT results indicated that catecholamine depletion abolished the analgesic activity of vortioxetine in all of the nociceptive tests (Figure 7). These findings together with the results of PCPA studies revealed that the central analgesic activity of vortioxetine is related to catecholamine and serotonin levels in the CNS.

The noradrenergic system modulates pain via α- and β-adrenergic receptors [23,41,42,43,44,45]. Therefore, we investigated the participation of adrenoceptors in the analgesic effect of vortioxetine. Our phentolamine studies showed that pre-treatment with this non-selective α-adrenoceptor blocker antagonised the analgesic effect of vortioxetine (Figure 8). These data indicate that α-adrenoceptors contribute to vortioxetine-induced analgesia. On the other hand, blocking β-adrenergic receptors with propranolol did not alter the analgesic effect (Figure 9), revealing that β-adrenoceptors do not mediate vortioxetine-induced analgesia, at least in healthy mice.

The involvement of opioid receptors in the analgesic effect of vortioxetine was examined using naloxone, a non-selective antagonist of opioid receptors. Naloxone pre-treatment eliminated the analgesic activity induced by 10 mg/kg vortioxetine in all of the nociceptive tests (Figure 10), suggesting that the opioidergic system mediates the acute analgesic effect of this drug at the supraspinal and spinal levels.

Vortioxetine has been reported to increase the levels of noradrenaline in various areas of brain [3]. Therefore, it is possible that this drug increase noradrenaline levels in pain-related regions of CNS, such as the descending noradrenergic pathway. The results of our AMPT studies pointing out the critical role of central noradrenaline level in the analgesic effect of vortioxetine are supportive for this idea. It has been shown that noradrenaline releasing from descending inhibitory pathway acting predominantly at the α_2_-adrenoceptors to induce analgesia [23]. Moreover, there is a well-described interaction between the opioidergic and α_2_ adrenergic receptors. Namely, co-administration of their agonists are known to produce synergistic analgesia, besides, α_2_ adrenoceptor agonists-induced analgesia can be reversed by naloxone administration [46]. In addition to the noradrenergic system, serotonergic system is known to interact with the opioid-mediated pain modulatory circuit [47]. It has been reported that the analgesic effects of µ and δ opioid receptor agonists at spinal and supraspinal levels were significantly weakened in Lmx1bf/f/p mice, which are genetically lack of central serotonergic neurons, while supraspinal analgesic efficacy of ĸ receptor agonists completely disappears. Central serotonergic system has been reported as a key component of supraspinal pain modulatory circuitry mediating opioid analgesia [48]. Our experimental data demonstrating the involvement of the serotonergic, noradrenergic, and opioidergic systems in vortioxetine-induced analgesia are consistent with these previous studies. Taken together, the analgesic effect of vortioxetine seems to arise because of interaction between the main endogenous pathways of the CNS, similar to its multimodal antidepressant effect.

One limitation of this study is that the antagonists used in the mechanism studies were administered in single doses and these agents have potential to act on other receptors than the targeted ones. For example, phentolamine, which shows its primary pharmacological effect by antagonizing α-adrenergic receptors, has also been reported to block serotonergic receptors and potassium channels and to inhibit histamine release from mast cells [49,50,51,52]. Moreover, NAN-190, a selective 5-HT_1A_ receptor antagonist, has been shown to block α_2_-adrenergic receptors in rodents [53]. Finally, the non-selective beta-adrenergic antagonist propranolol has been demonstrated to bind to serotonergic 5HT_1_ and 5HT_2_ receptors in brain membranes. Furthermore there is a pharmacological evidence that propranolol can behave as a 5-HT_1A_ receptor antagonist and a 5-HT_1B_ agonist in rat brain cortex [54]. Although the pharmacological significance of these activities is ambiguous, they may interfere with experimental results. Therefore, special attention was paid to dose selections and these agents were administrated in previously used doses [14,55,56,57,58,59,60,61,62,63,64,65], at which disappearance of the pharmacological effect have been associated to the involvement of targeted receptors in the activity. On the other hand, it is clear that further mechanical studies with various doses of antagonists would be useful to confirm the receptors that mediate the central analgesic effect of vortioxetine.

## 4. Materials and Methods

### 4.1. Animals

Adult male BALB/c mice (aged 12–15 weeks, body weight, 30–35 g) were used in this study. The animals were housed in a well-ventilated room at a controlled temperature of 24 ± 1 °C with a 12-h light/dark cycle (08:00–20:00). Water and food were provided ad libitum.

The animals were obtained from the Anadolu University Research Unit for Experimental Animals (Eskişehir, Turkey). The experimental protocol of the study approved by the Local Ethical Committee on Animal Experimentation of Anadolu University, Eskişehir, Turkey (Protocol code 2020-30 and date of approval: 14 July 2020). The relevant law of the Republic of Turkey (Regulation on the welfare and protection of animals used for experimental and other scientific purposes No. 28141, 15 February 2014) has been strictly followed.

### 4.2. Drugs and Administration Protocol

Vortioxetine hydrobromide (Brintellix^®^) was purchased from Lundbeck (North Ryde, NSW, Australia), while PCPA, NAN-190 hydrobromide, morphine sulphate (authorization date and number: 23 May 2018; 26/15), diazepam, AMPT, phentolamine hydrochloride, propranolol hydrochloride, and naloxone hydrochloride dehydrate were acquired from Sigma-Aldrich (St. Louis, MO, USA).

Vortioxetine, morphine sulphate and all the other chemicals, except AMPT and diazepam, were dissolved in physiological saline (0.9% NaCl) immediately before use. AMPT and diazepam was dissolved in saline with 10% Tween 80.

Mice were randomly assigned to the experimental groups. Randomisation was performed by an online software QuickCalcs (GraphPad Software, San Diego, CA, USA). Randomization, drug/agent administrations (Ü.K.), conduction of the experiments (N.T.Y.), outcome assessment and data analysis (Ö.D.C. and Ü.D.Ö.) were performed by the stated researchers. Investigators were blinded to group allocation during the conduction of the experiments, outcome assessment and data analysis.

Vortioxetine was orally administered to the mice at doses of 5, 10 and 20 mg/kg [15]. The reference drugs, morphine and diazepam, were administrated at 10 mg/kg (*i.p.*) and 2 mg/kg (*p.o.*) doses, respectively [14,66]. Experiments were initiated 30 min after the *i.p.* morphine injection and 60 min after the *p.o.* saline, diazepam and vortioxetine administrations.

For mechanistic studies, the mice were pre-treated with PCPA (serotonin-synthesis inhibitor, 100 mg/kg *i.p.* once a day, four consecutive days) [67], NAN-190 (serotonin 5-HT_1A_ receptor antagonist, 0.5 mg/kg, *i.p.*) [57,59], AMPT (catecholamine-synthesis inhibitor, 100 mg/kg, *i.p.*, 4 h prior to saline or vortioxetine administrations) [68], phentolamine (non-selective α-adrenoceptor blocker, 4 mg/kg, *i.p.*) [60], propranolol (non-selective β-adrenergic receptor blocker, 2 mg/kg, *i.p.*) [64] and naloxone (non-selective opioid receptor blocker, 5.48 mg/kg, *i.p.*) [14]. Except for PCPA and AMPT, the antagonists were administered 15 min before saline or vortioxetine administration.

**Figure 11:**
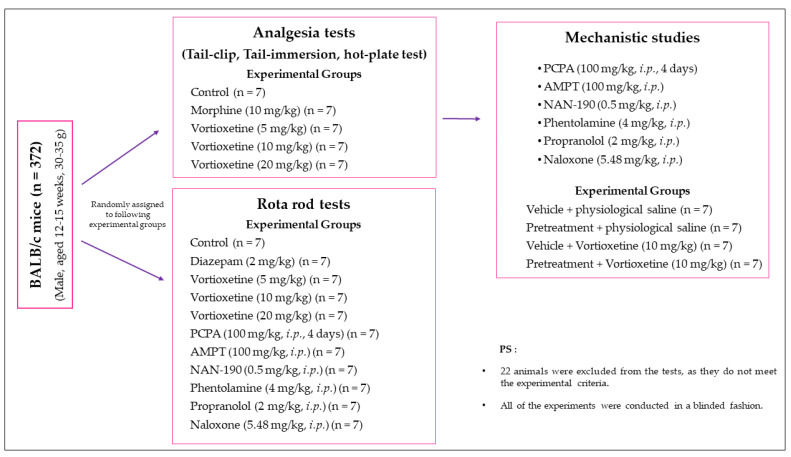
Brief summary of the experimental design.

The doses and ways of administration were chosen according to the previous experiences of our laboratory and to data previously reported for mice [14,15,57,59,60,64,67,68]. Details of the experimental settings and treatment protocols are presented in Figure 11.

### 4.3. Motor Coordination Analysis

#### Rota-Rod Test

Motor coordination of mice was evaluated using a Rota-rod device (Cat. no: 47600; Ugo Basile, Varese, Italy), as previously described [69]. The Rota-rod test is a two-stage test with training and experimental phases. In the training phase, the mice practised three times on the rotating rod, which was set at a constant speed of 16 rpm for three consecutive days. The mice that could not remain on the rod for more than 180 s were excluded from the experiments. In the experimental phase, the mice were placed on the rotating rod once more, and the falling time, a parameter of motor coordination, was automatically recorded. Diazepam was used as a reference drug [66].

### 4.4. Analgesic Activity Analysis

The analgesic activity of vortioxetine was investigated using the following acute nociception tests: tail-clip, tail-immersion and hot-plate tests.

#### 4.4.1. Tail-Clip Test

The tail-clip test was performed as previously described [14]. A metal artery clamp was applied to the tail of the mouse, and the latency of the nociceptive response (biting the clamp) was recorded using a stopwatch. A sensitivity test was performed before the experiments, and animals responding within 10 s were selected for the tests. Cut-off time was chosen as 10 s to avoid possible tissue damage.

#### 4.4.2. Tail-Immersion Test

The tail-immersion test was performed as described previously [18]. 1–2 cm of the tail of each mouse was immersed into hot water maintained at 52 ± 1 °C, and the latency of the nociceptive response (rapid flick of the tail) was recorded using a stopwatch. A sensitivity test was performed before the experiments, and animals responding within 4 s were selected for the tests. Cut-off time was chosen as 20 s to avoid possible tissue damage.

#### 4.4.3. Hot-Plate Test

The hot-plate test was performed as described earlier [14,70]. In this test, mice were individually placed on the aluminium plate of the hot-plate device (Cat. no: 7280; Ugo Basile, Varese, Italy), which was set at 55 ± 1.0 °C. Paw-licking, shaking or jumping latencies of each mouse were recorded. A sensitivity test was performed before the experiments and animals responding within 15 s were selected for the tests. Cut-off time was chosen as 30 s to avoid probable tissue damage.

In each of the nociceptive tests, response latencies of mice were recorded at 0th (pre-drug latency), 30th, 60th, 90th, 120th and 180th min. following the vehicle, reference drug and vortioxetine administrations to obtain time course of drug effects.

The following formula was used to convert tail-clip, tail-immersion, and hot-plate latencies to the percent maximum possible effect (MPE%):

MPE% = ((postdrug latency − predrug latency))/((cut off time − predrug latency)) × 100(1)

The MPE% values were plotted against time (0–180 min). Area under the MPE% versus time curves (AUC_0–180_) were calculated by using the GraphPad Prism ver. 8.4.3. (GraphPad Software, San Diego, CA, USA) based on the trapezoidal rule [18].

### 4.5. Statistical Analysis

The data used in statistical analyses were acquired from seven animals for each group. Variables were first investigated for normality and homogeneity of variance using Shapiro–Wilk and Levene tests, respectively. GraphPad Prism ver. 8.4.3 was used for statistical evaluations. Experimental data obtained from Rota-rod tests were analysed using one-way analysis of variance (ANOVA) followed by the Tukey’s honestly significant difference (HSD) test for multiple comparisons. Data acquired from time course nociceptive studies were evaluated by two-way repeated ANOVA, followed by the Tukey’s multiple comparisons tests. AUC_0–180_ data were analysed with one-way ANOVA followed by the Tukey’s HSD tests. Antagonist study results were evaluated by two-way ANOVA, followed by the Bonferroni test for multiple comparisons. Experimental results have been provided in terms of mean ± standard error of the mean. *p* < 0.05 was considered significant.

## 5. Conclusions

Findings of this research exhibited that vortioxetine exerts centrally mediated analgesic activity against acute mechanical and thermal nociceptive stimuli. Furthermore, this study demonstrated for the first time that serotonergic and catecholaminergic systems play critical roles in the vortioxetine-induced analgesia and that opioidergic, 5-HT_1A_ serotonergic and α-adrenergic receptors mediate this effect.

Since pain processing and modulation are highly complex functions regulated by various endogenous mechanisms in the nervous system, it may be beneficial to use more than one drug with different modes of action in pain clinics. This multi-drug analgesic approach can be advantageous due to the increased analgesic efficacy and reduced side effects. However, this time, problems related to patient compliance and drug-drug interaction risks may arise. On the other hand, inducing analgesia with a single drug acting on various pain pathways may make it possible to both strengthen the analgesic effect and avoid problems caused by polypharmacy [71,72]. Vortioxetine may meet these expectations as an analgesic drug with multimodal activity in the CNS [73]. However, further clinical studies are required to confirm this hypothesis.

## Data Availability

All relevant data are included within the manuscript. The raw data are available on request from the corresponding author.
